# 

**Published:** 1908-05

**Authors:** 


					﻿ImiT,
MAY, 1908.
No. 11.
TEXAS
MEDICAL
JOURNAL
“RED BACK”
PUBLISHED AT AUSTIN, TEXAS, BY
F. E. DANIEL, M. D.,	-	- Proprietor
PRINTED BY THE VON BOECKMANN-JONES CO.
Subscription, $1.00 a Year, in Advance. -	Single Copies, 10 Cents
Entered at the Postoffice at Austin, Texas, as Second-class Mail Matter
				

